# The complete mitochondrial genomes of two Chinese endemic cave fishes, *Sinocyclocheilus longibarbarus* and *Sinocyclocheilus punctatus* (Cypriniformes: Cyprinidae)

**DOI:** 10.1080/23802359.2021.1933635

**Published:** 2021-06-07

**Authors:** Fuguang Luo, Ruibin Yang, Jiahu Lan, Jie Huang, Zhiqiang Wan, Yanhong Wen

**Affiliations:** a Liuzhou Aquaculture Technology Extending Station, Liuzhou, China; b College of Fisheries, Huazhong Agricultural University, Wuhan, China; c Du’an Aquaculture Technology Extending Station, Hechi, China

**Keywords:** *Sinocyclocheilus*, cave fish, mitochondrial genome, phylogeny

## Abstract

*Sinocyclocheilus longibarbarus* and *Sinocyclocheilus punctatus* were collected from a karst cave Libo County, southwest of China. The two *Sinocyclocheilus* species can be distinguished obviously by external morphological characteristics. In this study, the complete mitochondrial genome sequences of two species were assembled, and both sequences reflected gene organization typical for mitochondrial DNA of the genus *Sinocyclocheilus,* comprising of 13 protein-coding genes (PCGs), 22 transfer RNA genes (tRNAs), 2 ribosomal RNA genes (rRNAs), and a large non-coding control region. Phylogenetic analysis showed that *S. punctatus* was first clustered together with *S. mutipunctatus*, and *S. longibarbarus* was closely related to *S. yishanensis.* The complete mitogenome of two species may provide useful information for the further taxonomic and phylogenetic studies.

*Sinocyclocheilus* genus is an endemic group in China; due to the isolation of cave environment, the species within the genus are strongly differentiated. To date, 75 species of effective species have been recorded, and most of the species live in caves and underground rivers. It is widely distributed in karst areas such as Yunnan, Guizhou and Guangxi. *Sinocyclocheilus longibarbarus* is only distributed in Guizhou (Wang and Chen 1989), while *Sinocyclocheilus punctatus* is distributed in Nandan, Huanjiang and Libo areas (Lan et al. 2017). Both of them belong to semi cave species with normal eyes and covered with scales, meanwhile, the two species can be distinguished by external morphological characteristics such as angular whisker, body shape, scale and body spot. Interestingly, the two species were collected from a karst cave in Libo County (23°14′15″N, 108°02′18″E), Guizhou Province, belonging to the Liujiang River System in the Pearl River Basin, and the specimen were fixed in 95% ethanol and preserved in Liuzhou Aquaculture Technology Extending Station, Liuzhou, China. Total genomic DNA were extracted from the fins through using traditional phenol-chloroform extraction method (Taggart et al. 1992). Then, we sequenced the complete mitochondrial genomes of the two *Sinocyclocheilus* species using the Illumina Hiseq4000 platform with *de novo* strategy (Tang et al. 2015) and submitted the genomes to GenBank with accession numbers MT361975 (*S. longibarbarus*) and MT361976 (*S. punctatus*), respectively.

The length of two complete circular mitogenomes were 16,570 bp with 44.08% GC content (*S. longibarbarus*) and 16,582 bp with 43.60% GC content (*S. punctatus*) respectively. Both genomes consisted of 13 protein-coding genes, 22 transfer RNAs, and 2 ribosomal RNAs, and the arrangement of all genes were identical to other *Sinocyclocheilus* species known to us (Wu et al. 2010; He et al. 2016; Luo et al. 2017; Peng et al. 2017). All genes of two *Sinocyclocheilus* species were located on the heavy strand (H-strand) except for ND6 gene and eight tRNA genes, and the protein-coding genes started with a traditional ATG except for COX1, which started with one infrequent GTG instead, and terminated with stop codons TAA, TAG, or a single T-base. The control region (D-loop) of two *Sinocyclocheilus* species were located between tRNA-Phe and tRNA-Pro, with 920 bp (*S. longibarbarus*) and 928 bp in length (*S. punctatus*), respectively.

To determine the phylogenetic relationship of both species in the *Sinocyclocheilus* family, a Bayesian Inference (BI) tree was constructed on MrBayes v3.2.7 using the 13 PCGs in mitogenomes of 17 species. *Danio rerio* and *Cyprinus carpio* was set as outgroups. We obtained the best-fit substiution model (GTR + I + G) by MrModelTest 2.3 (Nylander 2004) in PAUP 4.0, and Four parallel runs of Markov Chain Monte Carlo (MCMC) were run for 1,000,000 generations, sampling every 1000 generations and discarded 100 trees as burn-in. Phylogenetic analysis showed that *S. punctatus* was not first clustered together with *S. longibarbarus* which lived in the same cave but *S. mutipunctatus*, and *S. longibarbarus* was closely related to *S. yishanensis* (Figure 1), it suggested that long-term reproductive isolation might lead to species differentiation, while convergent evolution had little effect on species differentiation in similar environments.

**Figure 1.**
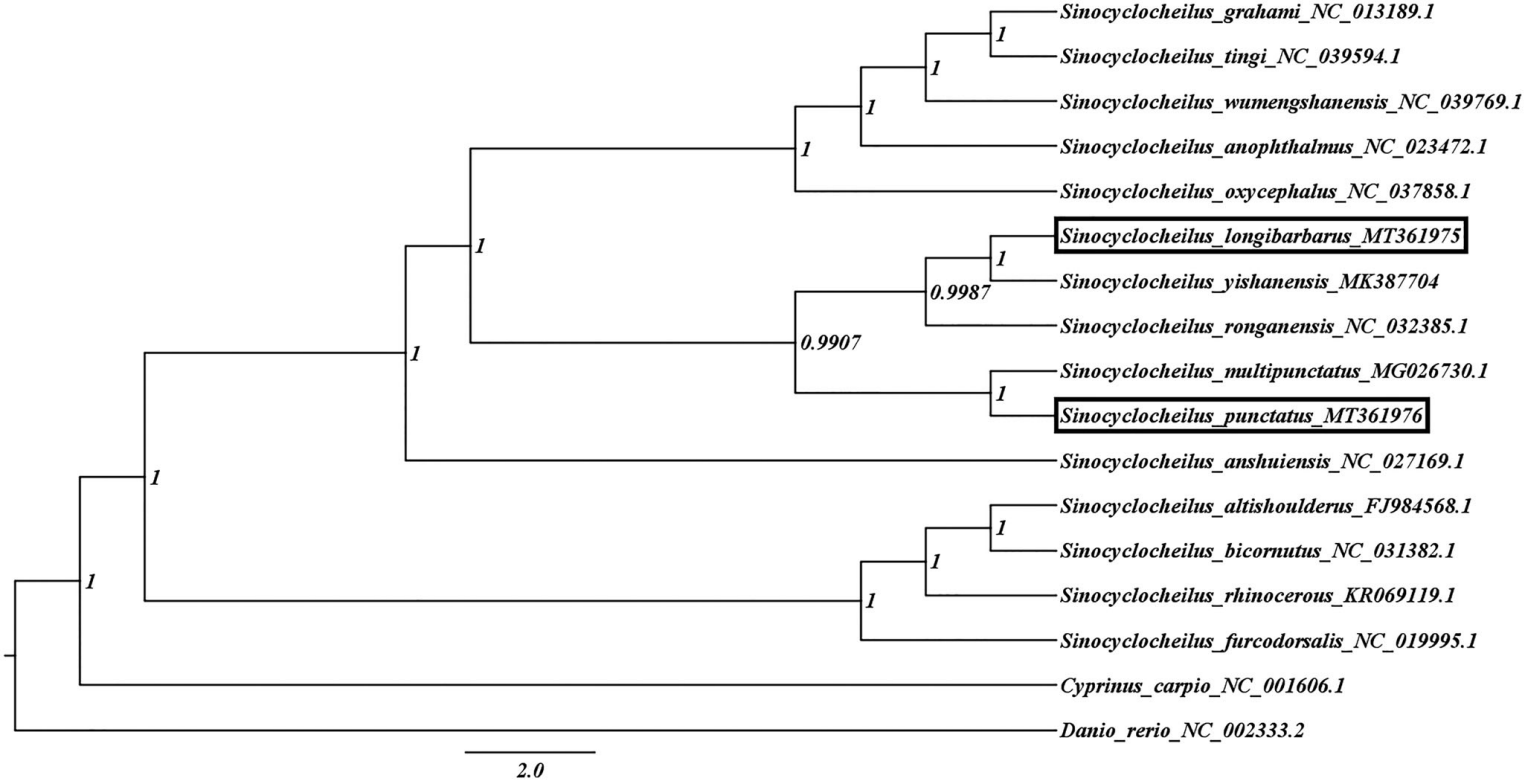
Bayesian phylogenetic tree based on 13 mitochondrial protein-coding genes of *S. longibarbarus*, *S. punctatus* and other 15 affinis fishes (GenBank accession numbers provided). *Danio rerio* and *Cyprinus carpio* were included as the outgroup taxon.

## Data Availability

The data that support the findings of this study are openly available in GenBank of NCBI at https://www.ncbi.nlm.nih.gov, reference number MT361975 (*Sinocyclocheilus longibarbarus*) and MT361976 (*Sinocyclocheilus longibarbarus*).
